# Identification of Prognostic Stromal-Immune Score–Based Genes in Hepatocellular Carcinoma Microenvironment

**DOI:** 10.3389/fgene.2021.625236

**Published:** 2021-02-11

**Authors:** Shanshan Liu, Guangchuang Yu, Li Liu, Xuejing Zou, Lang Zhou, Erqiang Hu, Yang Song

**Affiliations:** ^1^Country Guangdong Provincial Key Laboratory of Viral Hepatitis Research, Hepatology Unit and Department of Infectious Diseases, Nanfang Hospital, Southern Medical University, Guangzhou, China; ^2^State Key Laboratory of Organ Failure Research, Nanfang Hospital, Southern Medical University, Guangzhou, China; ^3^Department of Bioinformatics, School of Basic Medical Sciences, Southern Medical University, Guangzhou, China; ^4^Department of Medical Quality Management, Nanfang Hospital, Southern Medical University, Guangzhou, China; ^5^Department of Radiation Oncology, Nanfang Hospital, Southern Medical University, Guangzhou, China

**Keywords:** liver cancer, ESTIMATE, bioinformatics analysis, biomarker, tumor-microenvironment

## Abstract

A growing amount of evidence has suggested the clinical importance of stromal and immune cells in the liver cancer microenvironment. However, reliable prognostic signatures based on assessments of stromal and immune components have not been well-established. This study aimed to identify stromal-immune score–based potential prognostic biomarkers for hepatocellular carcinoma. Stromal and immune scores were estimated from transcriptomic profiles of a liver cancer cohort from The Cancer Genome Atlas using the ESTIMATE (Estimation of STromal and Immune cells in MAlignant Tumors using Expression data) algorithm. Least absolute shrinkage and selection operator (LASSO) algorithm was applied to select prognostic genes. Favorable overall survivals and progression-free interval were found in patients with high stromal score and immune score, and 828 differentially expressed genes were identified. Functional enrichment analysis and protein–protein interaction networks further showed that these genes mainly participated in immune response, extracellular matrix, and cell adhesion. *MMP9* (matrix metallopeptidase 9) was identified as a prognostic tumor microenvironment–associated gene by using LASSO and TIMER (Tumor IMmune Estimation Resource) algorithms and was found to be positively correlated with immunosuppressive molecules and drug response.

## Introduction

Hepatocellular carcinoma (HCC) is the third leading cause of cancer death worldwide. The median survival of HCC patients in China is about 23 months, and ≥ 60% of patients present with intermediate-stage or advanced-stage HCC (Kanwal and Singal, 2019; Yang et al., 2019). Currently, the main treatment for HCC patients in early stages is surgery, combination with transarterial chemoembolization, ablation, and liver transplantation. For others in advanced stages, the effective approaches involve molecular targeting agents (sorafenib, lenvatinib, and regorafenib). Although these methods have improved the prognosis of HCC patients, the overall survival (OS) of HCC remains challenging for the heterogeneity of HCC. And also, there is still a lack of molecular markers used in determination of prognosis and treatment for patients (Bruix et al., 2016).

The liver cancer microenvironment consists of not only tumor cells but also stromal cells, including distinct immune cell subsets. Tumor-infiltrating immune cells and stromal cells are associated with angiogenesis, immune suppression, chemotherapeutic resistance, and tumor cell migration (Affo et al., 2017; Barry et al., 2020; Jin and Jin, 2020; Son et al., 2020; Zhang et al., 2020). An increasing amount of evidence has suggested the clinical importance of stromal cells and immune cells in the microenvironment of liver cancer tissues, tumor microenvironment (TME)–associated genes also have potential as novel biomarkers for a range of cancers (Yang et al., 2020).

In the present study, the Estimation of STromal and Immune cells in MAlignant Tumors using Expression data (ESTIMATE) algorithm (Yoshihara et al., 2013) was applied to estimate the stromal and immune scores of a series of cancer tissues based on their transcriptional profiles, to perform a comprehensive analysis of immune and stromal cells, and to correlate the data to clinical outcomes of patients.

The least absolute shrinkage and selection operator (LASSO) method is a compressed estimation used to obtain a refined model by constructing a penalty function (Korenberg, 2006). It can help with the selection of variables at the time of parameter estimation so as to better solve the multicollinearity problem of regression analysis. A growing body of research confirms that LASSO is an effective method for gene selection of tumors (Wang et al., 2020; Xu et al., 2020).

Tumor IMmune Estimation Resource (TIMER) integrates multiple state-of-the-art algorithms for immune infiltration estimation, which can explore various associations between immune infiltrates and genetic features in The Cancer Genome Atlas (TCGA) cohorts (Li et al., 2017, 2020). Computational Analysis of REsistance (CARE) is a computational method focused on targeted therapies, to infer genome-wide transcriptomic signatures of drug efficacy from cell line compound screens (Jiang et al., 2018). Previous studies have confirmed that the efficacy of immunotherapy is strongly influenced by the composition and abundance of immune cells in the TME (Boyero et al., 2020).

Thus, we combined LASSO, TIMER algorithms, and CARE to preliminarily demonstrate that the expression of TME-associated genes could be new prognostic and reliable drug response biomarkers for HCC patients.

## Materials and Methods

### Database

In total, data from 365 HCC patients and 18,161 RNAs extracted from RNA-seq data according to ENSEMBL Genomes (hg38) were analyzed in this study. All RNA expression data and the corresponding clinical data were obtained from TCGA (data version, July 19, 2019)^1^. The clinicopathological characteristics of the analyzed patients are listed in Supplementary Table 1. The progression-free interval (PFI) is characterized as a time without a new tumor occurrence or a death from cancer. The Estimation of STromal and Immune cells in MAlignant Tumors using Expression data (ESTIMATE) algorithm was applied to the normalized expression matrix for estimating the stromal and immune scores by using “estimate” R package in R software (version: 3.6.3) for each HCC sample.

### Correlations Between Prognoses and Stromal/Immune Scores

OS and PFI was used as the primary prognosis endpoint and was estimated by the GraphPad Prism 8.0. Supplementary Figure 3B is realized by R package “Survival” (Therneau, 2020), “Survminer” (Kassambara et al., 2019), and “timeROC” (Paul Blanche and Jacqmin-Gadda, 2013). Based on the stromal and immune scores estimated from each sample, patients were classified into two groups by using X-tile, and prognoses for each group were examined. The bioinformatics tool, X-tile (Camp et al., 2004), was used to determine the optimum cutoff point according to the minimum *P*-value defined by the Kaplan–Meier analysis and log-rank test. The principle of X-tile is “enumeration method that different values are grouped as truncation values to conduct statistical tests, and the test result with the lowest *P*-value can be considered as the best truncation value. The survival outcomes of the two groups were compared by log-rank tests. *P* < 0.05 was considered as statistically significant.

### Identification of Differentially Expressed Genes

Data analysis was performed using an open-source web tool NetworkAnalyst^2^ (Xia et al., 2013a,b; Zhou et al., 2019). Log2 fold change > 1 and adjusted *P* < 0.05 were set as the cutoffs to screen for differentially expressed genes (DEGs). A website Venn diagrams tool (Bardou et al., 2014)^3^ was used to identify the commonly upregulated or downregulated DEGs in the immune and stromal groups. Heatmaps and clustering were generated using the R package “ggplot2” (Wickham, 2016), “ggtree” (Yu, 2020b), and “aplot” (Yu, 2020a).

### Gene Ontology and Kyoto Encyclopedia of Genes and Genomes Pathway Enrichment Analyses

GO (Gene Ontology) enrichment analyses were performed by the “Goseq” (Young et al., 2010) R package, and visualization of bubble diagrams used Hiplot^4^. KEGG (Kyoto Encyclopedia of Genes and Genomes) enrichment analyses and visualization of intersection genes were performed by the “clusterProfiler” (Yu et al., 2012) R package and “enrichplot” (Yu, 2019) R package with *P* < 0.05 as the cutoff value.

### Protein–Protein Interaction Network Construction

The protein–protein interaction (PPI) network was retrieved from Search Tool for the Retrieval of Interaction Gene/Proteins (STRING) (Szklarczyk et al., 2019) database with high confidence (0.7) and reconstructed via the Cytoscape software (Shannon et al., 2003). In Cytoscape, we used Molecular COmplex DEtection (MCODE) (Bader and Hogue, 2003) to select two clusters that contained the largest number of nodes. ClueGo (Bindea et al., 2009) App was used to perform enrichment analysis of each cluster selected by MCODE.

### Identification of TME-Associated Prognostic Genes

LASSO algorithm was used to identify candidate genes by “glmnet” (Friedman et al., 2010) R package with the number of lambda = 1,000. Clinical outcomes and gene expression profiles were analyzed by LASSO. Lambda.min is the cutoff point that brings minimum mean cross-validated error. Genes with the highest lambda values were selected for further analysis.

### Identification of TME-Associated Prognostic Genes

The TIMER algorithm was used to calculate the tumor abundance of six infiltrating immune cells (CD4^+^ T cells, CD8^+^ T cells, B cells, neutrophils, macrophages, and dendritic cells) based on RNA-Seq expression profiles data. The correlation between the selected prognostic genes and immune cells was calculated by Spearman correlation analysis by TIMER. The estimation results were calculated by TIMER2.0, CIBERSORT, quanTIseq, xCell, MCP-counter, and EPIC methods. Relations between immunoinhibitors and expression of matrix metallopeptidase 9 (*MMP9*) were calculated by Spearman correlation analysis by a web tool TISIDB^5^ (Ru et al., 2019). The correlation coefficient value <0.3 indicates that the correlation is negligible, whereas the correlation coefficient ≥ 0.3 indicates a positive/negative correlation. The CARE software^6^ was used to identify genome-scale biomarkers of targeted therapy response using compound screen data. For each gene, the CARE score indicates the association between its molecular alteration and drug efficacy. A positive score indicates a higher expression value (or presence of mutation) to be associated with drug response, whereas a negative score indicates drug resistance.

### Statistical Analysis

Unpaired *t*-test was used to compare two groups of continuously distributed variables. Jonckheere–Terpstra test was used to compare three or more groups of continuously distributed variables. The FDR correction was performed in multiple tests. ^∗^*P* < 0.05, ^∗∗^*P* < 0.01, ^∗∗∗^*P* < 0.001.

## Results

### Association of Stromal and Immune Scores With HCC Pathology and Prognosis

**FIGURE 1:**
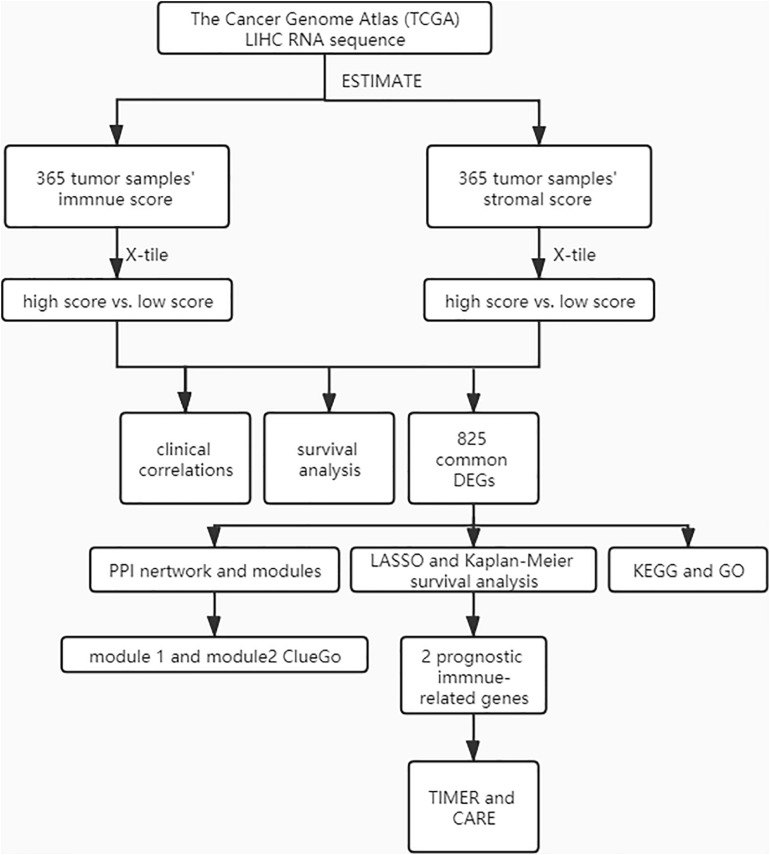
The general pipeline of the data analysis protocol.

A cohort containing 365 liver hepatocellular carcinoma patients with available expression data and clinical information in TCGA database was analyzed. The general pipeline of the data analysis protocol is shown in Figure 1, and the links of tools are listed in Supplementary Table 6. The clinicopathological characteristics of the analyzed patients are listed in Supplementary Table 1. Based on the gene expression data, immune and stromal scores were calculated using the ESTIMATE algorithm (Supplementary Table 2). The associations of stromal and immune scores with HCC patient pathological characteristics were examined by comparing the score distributions among different tumor stages and differentiation grades.

**FIGURE 2:**
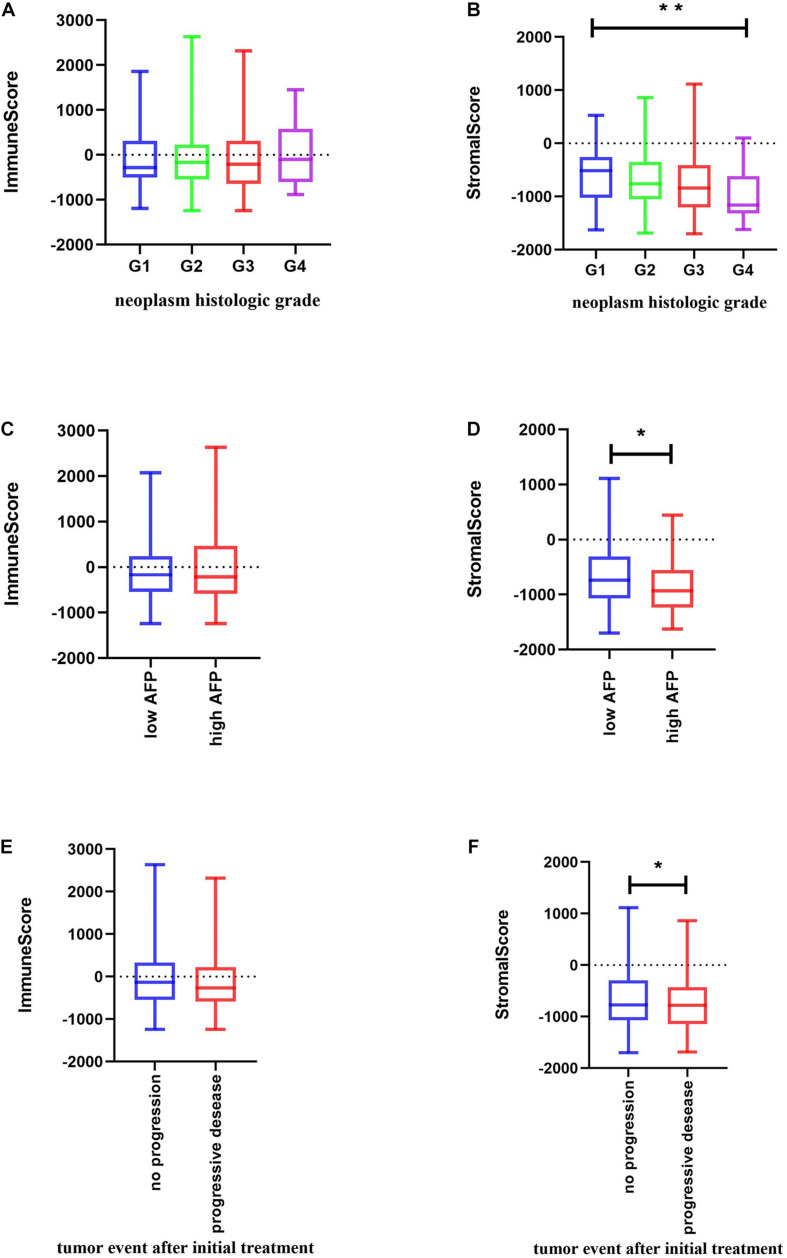
Relationship between immune and stromal scores and HCC clinical and pathological data. **(A,B)** Distribution of immune and stromal scores of HCC grades. **(C,D)** Distribution of immune and stromal scores of AFP value of HCC. AFP is divided into high and low groups at the limit of 400 ng/mL. **(E,F)** Distribution of immune and stromal scores of new tumor event after initial treatment of HCC. Unpaired *t*-test was used to compare two groups of continuously distributed variables. Jonckheere–Terpstra test was used to compare three or more groups of continuously distributed variables. ^∗^*P* < 0.05 and ^∗∗^*P* < 0.01.

Significant associations were observed between stromal scores and tumor differentiation grades; tumors with poorer differentiation yielded higher stromal scores than those differentiated well (Jonckheere–Terpstra test, *P* = 0.002) (Figures 2A,B).

As previously described, serum α-fetoprotein (AFP) values are not only of diagnostic value but also of prognostic significance in patients with HCC (Galle et al., 2019). Thus, we compared changes in immune and stromal scores between AFP low (AFP ≤ 400 ng/mL) and high (AFP > 400 ng/mL) samples. The AFP high cases had the lowest stromal scores (unpaired *t*-test, *P* = 0.0204) (Figure 2D). Evidence suggests that AFP plays an immune-suppressing role (Yang et al., 2018), but we found that there is no significant difference in the immune score as shown in Figure 2C. We further used the TIMER algorithm to evaluate the effect of AFP on the immune infiltration of HCC, and results showed that the expression of AFP was weakly correlated with the infiltration abundance of the six immune cells (Supplementary Figure 1A). AFP is dynamic in the occurrence and development of HCC, whereas TCGA patients were only tested for AFP at the time of initial diagnosis, which may lead to the bias of the results in our study.

Also, when we compared the immune and stromal scores between patients with a new tumor event and without new tumor event after initial treatment, patients without a new tumor event had higher immune and stromal scores (unpaired *t*-test, *P* = 0.0461 for stromal score and *p* = 0.1966 for immune score) (Figures 2E,F).

We also analyzed the correlation between other clinical factors and the immune profile, but found no statistically significant difference (Supplementary Figures 1B,C).

**FIGURE 3:**
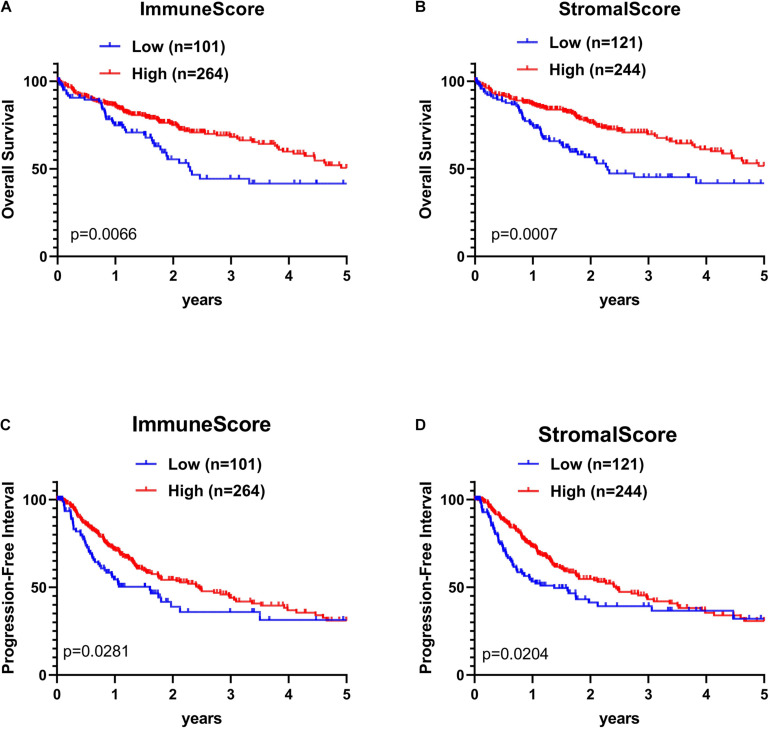
Kaplan–Meier (KM) survival curve of HCC patients based on their immune-stromal scores. Patients were classified into high immune-stromal scores groups and low immune/stromal scores groups by using X-tile. **(A)** The KM curve of overall survival (OS) time of high and low immune score group. **(B)** The KM curve of OS time of high and low stromal score group. **(C)** The KM curve of PFI time according to immune scores. **(D)** The KM curve of progression-free interval (PFI) time according to stromal scores. The survival outcomes of the two groups were compared by log-rank tests. *P* < 0.05 was statistically significant.

The association of stromal and immune scores with HCC prognosis was evaluated by dividing patients optimally into two groups based on their scores by using X-tile (see section “Materials and Methods” for details). We found that the high immune score and stromal score positively correlated with both OS and PFI (Figures 3A–D).

### Comparison of Gene Expression Profile With Immune Scores and Stromal Scores in HCC

**FIGURE 4:**
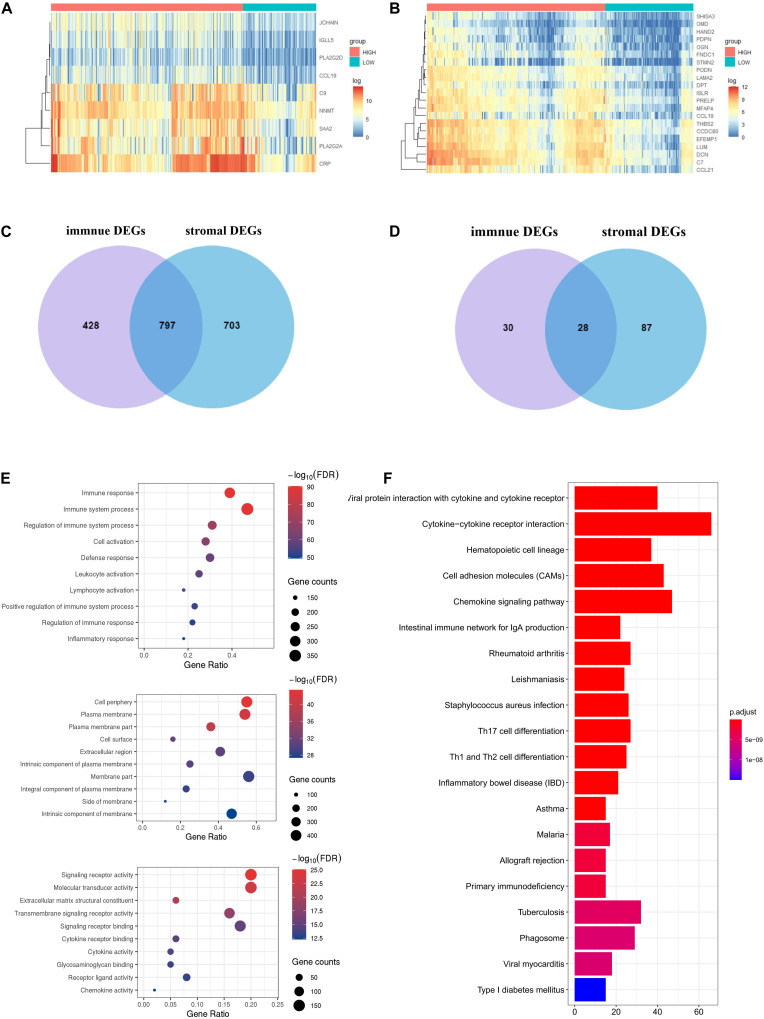
Expression profiles and biological functions of stromal and immune score–related DEGs. **(A,B)** Heatmaps showing expression profiles for selected stromal score (right) and immune score (left)–related DEGs (Log2 fold change ≥ 3 and adjusted *P* < 0.05) with unsupervised hierarchical clustering analyses, using the complete linkage method to measure distances between clusters. **(C)** Shows the commonly upregulated DEGs, and **(D)** shows the commonly downregulated DEGs. **(E)** The top 10 of biological processes GO terms (top), cellular component GO terms (middle), and molecular function GO terms (bottom); **(F)** KEGG (Kyoto Encyclopedia of Genes and Genomes) analysis of microenvironment-related DEGs.

To identify the immune-related and stromal-related genes, differential analysis by using NetworkAnalyst was performed (Supplementary Table 3). The expression profiles of stromal and immune score–related DEGs are visualized, respectively, on the heatmaps (Figures 4A,B).

There were 797 shared DEGs overexpressed in both the stromal score and immune score groups (Figure 4C), and a total of 28 common DEGs were found to be underexpressed in both the stromal score and immune score groups (Figure 4D). Eight hundred twenty-five intersection genes were selected for further analysis (overlap zone in Figures 4C,D).

Using the “Goseq” and “clusterProfiler” R packages, 1,371 GO terms and 73 KEGG terms were indicated (Supplementary Table 4).

The results showed the top 10 biological processes GO terms, cellular component GO terms, and molecular function GO terms (Figure 4E). The correlation between the intersection genes and the top five biological processes is shown in Supplementary Figure 2A. The top 20 KEGG analysis showed that the intersection genes were associated with immune responses (Figure 4F).

### Protein–Protein Interactions Among Intersection Genes

**FIGURE 5:**
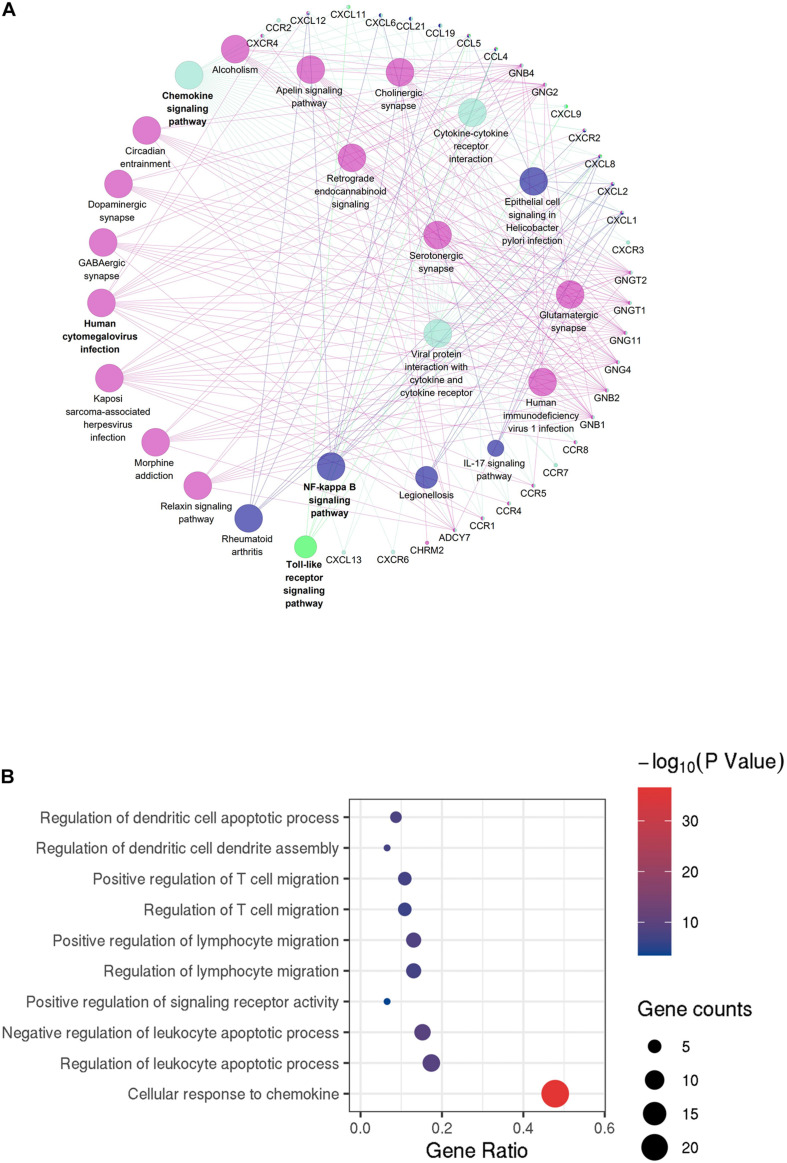
Protein–protein interaction (PPI) network of microenvironment-related genes. **(A)** Module 1 is the top module in the PPI network. **(B)** GO analyses of module 1 (top 10 of biological processes GO terms). The color and thickness of edges reflect the combined score.

To better understand the interplay among the identified DEGs, we obtained PPI networks using the STRING tool. Using the MCODE software, we found modules in the network; the network was made up of eight modules, which included 408 nodes and 2,702 edges. We selected the top two significant modules for further analysis (Figure 5A and Supplementary Figure 2B).

GO analyses of module 1 (Figure 5A) by ClueGo are shown in Figure 5B. Likewise, GO analyses of module 2 (Supplementary Figure 2B) by ClueGo are shown in Supplementary Figure 2C. The results demonstrated that module 1 was mainly enriched in regulation of dendritic cell apoptotic process, regulation of dendritic cell dendrite assembly, and positive regulation of T cell migration. Module 2 was mainly enriched in the regulation of phospholipase C activity, cellular response to interferon-γ (IFN-γ) and IFN-γ–mediated signaling pathway. Obviously, the top two modules were enriched for functional terms related to immune response processes, especially T cell responses.

### Identification of Prognostic DEGs in HCC

**FIGURE 6:**
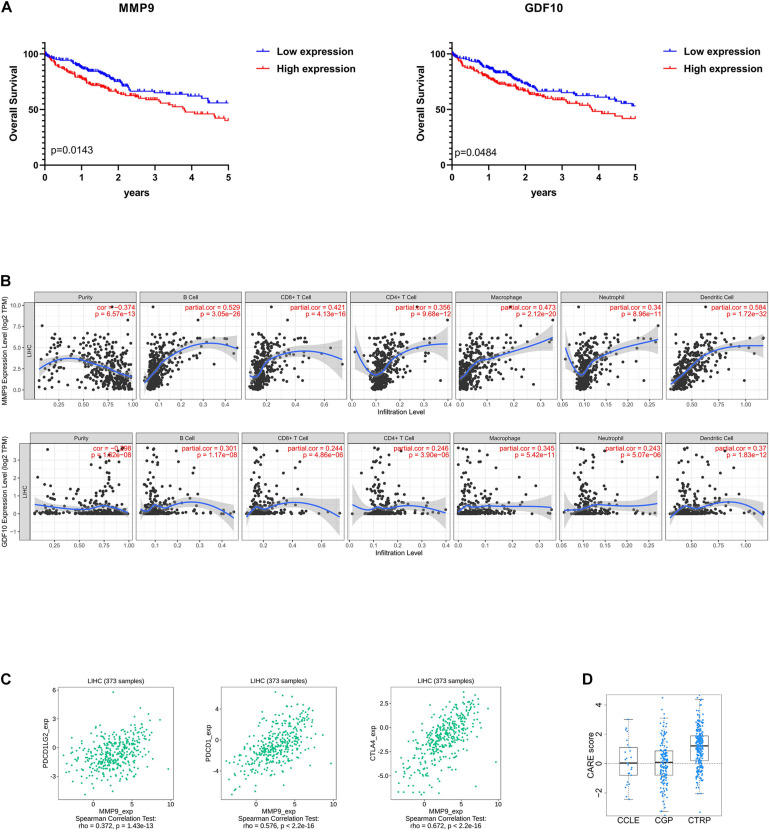
Selection of microenvironment-related prognostic genes and the analysis of immune cell infiltration and immunoinhibitor. **(A)** Kaplan–Meier (KM) survival curve of *GDF10* and *MMP9*. Patients were divided into two groups based on the median of gene expression. The survival outcomes of the two groups were compared by log-rank tests. *P* < 0.05 was statistically significant. **(B)** Correlation of microenvironment-related prognostic genes’ expression with immune infiltration level. **(C)** Relations between three kinds of immunoinhibitors and expression of *MMP9*. *P* < 0.05 was statistically significant, and partial correlation ≥0.3 indicates strong correlation. **(D)** The CARE score of *MMP9* on CCLE, CGP, CTRP dataset. A positive score indicates a higher expression value to be associated with drug response.

To enrich for genes with the greatest prognostic values, we performed LASSO algorithm, and seven genes were identified (Supplementary Figure 3A). We also analyzed the association between the seven genes and OS using the Kaplan–Meier survival analysis. We found that the high levels of *GDF10* (*P* = 0.0484) and *MMP9* (*P* = 0.0143) negatively correlated with OS (Figure 6A).

### Immune Cell Infiltration Analysis

To determine whether there is a correlation between tumor infiltration with immune cells and immune-related gene expression, the tumor infiltration with multiple immune cells was analyzed by TIMER 2.0 and other methods (Supplementary Table 5). Figure 6B shows the strong correlation between six types of immune cell infiltration and the expression of *MMP9*. The expression of *MMP9* positively correlated with the infiltrating levels of B cells (partial correlation = 0.529, *P* = 3.05e-26), CD8^+^ T cells (partial correlation = 0.421, *P* = 4.13e-16), CD4^+^ T cells (partial correlation = 0.356, *P* = 9.68e-12), macrophages (partial correlation = 0.473, *P* = 2.12e-20), neutrophils (partial correlation = 0.34, *P* = 8.96e-11), and dendritic cells (partial correlation = 0.584, *P* = 1.72e-32). GDF10 expression was weakly associated with different immune cell infiltrates (Figure 6B).

We analyzed the correlation between *MMP9* and immune checkpoints in liver cancer. MMP9 was found to be correlated with the expression of a series of immune checkpoints. Particularly, MMP9 was significantly correlated with PDCD1 (ρ = 0.576), PDCD1LG2 (ρ = 0.372), and CTLA4 (ρ = 0.672) (Figure 6C).

Besides, identifying reliable drug response biomarkers is a significant challenge in cancer research. We present CARE, a computational method that enables large-scale inference of response biomarkers and drug combinations for targeted therapies using compound screen data. High expression of *MMP9* has been associated with better response to immunotherapies on CTRP dataset (Figure 6D).

## Discussion

Prognosis prediction for liver cancer patients remains challenging for clinicians and investigators. Through a specific view of the microenvironment, this study provides a stromal-immune score–based gene signature to help answer this important clinical question.

Using the ESTIMATE algorithm, we revealed the correlation between the immune-stromal scores and the clinical HCC characteristics obtained from TCGA-CDR. The stromal and immune scores for tumor tissue were found to be positively associated with the clinicopathologic characteristics of the tumor and the patient’s prognosis. By analyzing the correlation between the immune scores and tumor recurrence, our data show that high-immune-score patients have a longer PFI and OS rates, indicating that the TME composition affects the clinical outcomes of HCC patients, which is consistent with previous studies (Haider et al., 2020).

Next, we analyzed 825 DEGs yielded from a comparison of high- versus low-immune-score (or stromal scores) groups and found that many of them were involved in the TME, specifically regulate T cell functions (Figure 4E). This is consistent with previous reports that the functions of immune cells and extracellular matrix molecules are interrelated in building TME in HCC (Lu et al., 2019; Yin et al., 2019). Moreover, we were able to construct two PPI modules (Figure 5 and Supplementary Figures 2B,C), the major of which were related to IFN-γ. We infer that these TME-associated genes might affect the development of HCC by affecting the T cell functions.

Finally, by using the LASSO algorithm (Supplementary Figure 3A), we identified seven TME-related genes. Of the seven genes identified, high levels of *GDF10* and *MMP9* showed a negative correlation to OS, which has been reported to be involved in carcinogenesis and the development of various cancers (Chang et al., 2017; Reggiani et al., 2017; Tekin et al., 2020a). We further correlated the degree of infiltration of six immune cell types with the expression of GDF10 and MMP9 by using TIMER algorithm. The expression of *MMP9* was positively associated with the abundance of six immune in tumor tissues. It is worth reminding that our results did not contradict previous findings that high infiltration of CD8^+^ T cells indicated beneficial prognosis, but extended and enriched this conclusion. In the recent literature, tumor with higher CD8^+^ T cell infiltration, but T cell dysfunction and increased immune escape result in a poor prognosis (Hossain et al., 2020; Saka et al., 2020).

Prior studies have largely focused on *MMP*s’ ability to promote the invasion and metastasis of cancer cells (Nart et al., 2010; Chen et al., 2012), while evidence is mounting that *MMP*s are highly associated with the microenvironment of tumors and immune cells (Kessenbrock et al., 2010; Li et al., 2016). For example, *MMP9*-cleaved osteopontin fragments contribute to tumor immune escape by inducing the expansion of myeloid-derived suppressor cells (Shao et al., 2017). Macrophages secrete *MMP9* to induce mesenchymal transition, which supports the tumor-promoting role of macrophage influx (Tekin et al., 2020b). Besides, *MMP9* is associated with neutrophil migration (Koymans et al., 2016). Our study confirms the above conclusions and has found that *MMP9* might associate with T cell dysfunction, despite high CD8^+^ cytotoxic T lymphocyte infiltration.

In addition, we also observed that high expression of *MMP9* indicated higher levels of immune inhibitors (immune checkpoints), better response to immunotherapies, and poor survival in partial HCC patients, which was in line with our above analysis that some HCC patients with high CD8^+^ T cell infiltration but with dysfunction were immunosuppressed. And previously, inhibition of *MMP9* could modulate immunosuppression in tumor (Melani et al., 2007). We also compared the prediction effect between the other factors, such as AFP (Supplementary Figure 1A) and programmed cell death protein 1 (PDCD1) (Supplementary Figure 3B), whereas AFP is not a good predictor of the abundance of immune invasion in HCC tissues, and PDCD1 is weakly correlated with the prognosis of HCC. Hence, *MMP9* may be an effective biomarker to evaluate the immune status of patients and predict the effectiveness of immunotherapy before treatment. However, this conclusion will need to be confirmed by clinical trials in the future.

In summary, from comprehensively analyzing the correlation between microenvironmental and genetic factors of TCGA database applied by ESTIMATE algorithm-based immune and stromal scores, we identified *MMP9* as a potential TME-related biomarker of prognostic and immunotherapy response. However, because of the lack of large sequenced HCC cohort and prospective clinical trials that have received immunotherapy, the effect of *MMP9* expression on the efficiency of immunotherapy in HCC patients remains concerned.

## Data Availability Statement

The original contributions presented in the study are included in the article/Supplementary Material, further inquiries can be directed to the corresponding author/s.

## Author Contributions

SL, GY, and LL: conception and design of study. SL: acquisition of data and drafting the manuscript. SL, XZ, LZ, EH, and YS: analysis and interpretation of data. XZ, GY, and LL: revising the manuscript critically for important intellectual content. SL, GY, LL, XZ, LZ, EH, and YS: approval of the version of the manuscript to be published. All authors contributed to the article and approved the submitted version.

## Conflict of Interest

The authors declare that the research was conducted in the absence of any commercial or financial relationships that could be construed as a potential conflict of interest.

## Footnotes

1 https://xenabrowser.net

2 https://www.networkanalyst.ca/NetworkAnalyst/home.xhtml

3 http://www.ehbio.com/test/venn/#/

4 https://hiplot.com.cn

5 http://cis.hku.hk/TISIDB/

6 http://care.dfci.harvard.edu/
