# Supplementary material for: Identification of Prognostic Stromal-Immune Score–Based Genes in Hepatocellular Carcinoma Microenvironment
Source: Front Genet. 2021 Feb 11;12:625236. doi: 10.3389/fgene.2021.625236 (PMC7905188; doi:10.3389/fgene.2021.625236)
Supplement: Supplementary file 1 [file Data_Sheet_1.DOCX]

Supplementary Material

# Supplementary Tables

**Supplementary Table 1.** The clinical data of LIHC.

**Supplementary Table 2.** The immune scores and stromal scores of LIHC.

**Supplementary Table 3.** Differentially expressed genes selected based on immnue scores.

**Supplementary Table 4.** GO terms and KEGG terms of differentially expressed genes.

**Supplementary Table 5.** The infiltration of immune cells of HCC patients.

**Supplementary Table 6.** The links of softwares and cohort.

# Supplementary Figures

**Supplementary Figure 1.** The correlation between clinical factors and the immune profile. **(A)**Correlation of AFP expression with immune infiltration level. **(B)**Distribution of immune and stromal scores of NAFLD/NASH, HCV/HBV infection, gender, and age of HCC. **(C)**Correlation of gender and age with immune infiltration level. Age is divided by median. p < 0.05 was considered as statistically significant and partial.cor≥ 0.3 indicates strong correlation.

**Supplementary Figure 2. (A)**The correlation between the intersection genes and the top 5 biological processes. **(B)**module 2 in the PPI network. **(C)** GO analyses of module 2(top 10 of biological processes GO terms). The color and thickness of edges reflect the combine score.

**Supplementary Figure 3. (A)**Lasso (Least Absolute Shrinkage and Selector Operation) algorithms were preformed to further select microenvironment related prognostic genes. **(B)** Kaplan-Meier survival analysis of PDCD1 and Time-dependent ROC analysis the of PDCD1. ROC receiver operating characteristic. For Kaplan–Meier curves, p-values and hazard ratio (HR) with 95% confidence interval (CI) were generated by log-rank tests and univariate Cox proportional hazards regression. p < 0.05 was considered as statistically significant.
