# Supplementary figures and images for: Identification of Prognostic Stromal-Immune Score–Based Genes in Hepatocellular Carcinoma Microenvironment
Source: Front Genet. 2021 Feb 11;12:625236. doi: 10.3389/fgene.2021.625236 (PMC7905188; doi:10.3389/fgene.2021.625236)

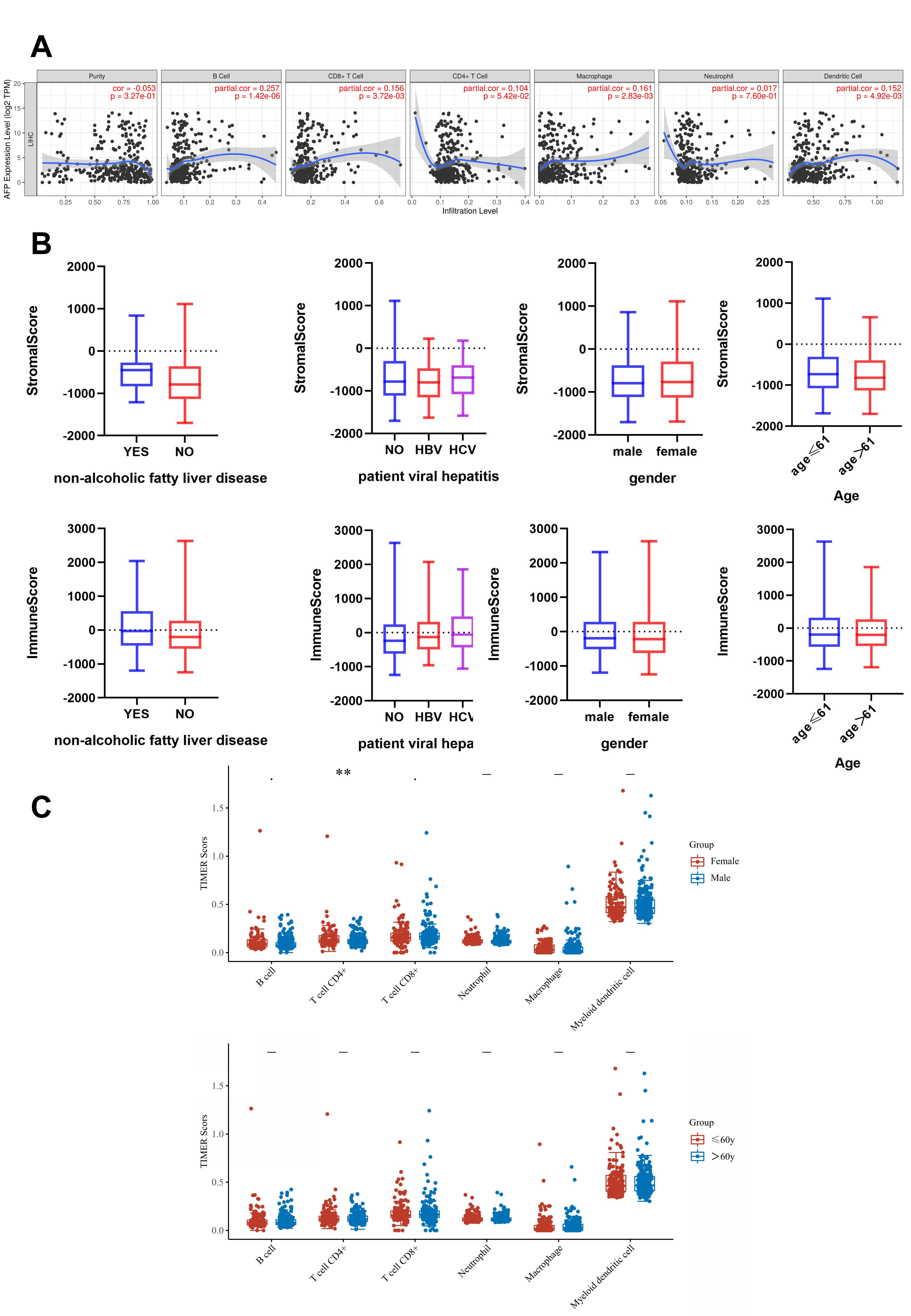

Supplement: Supplementary file 2 [file Image_1.JPEG]

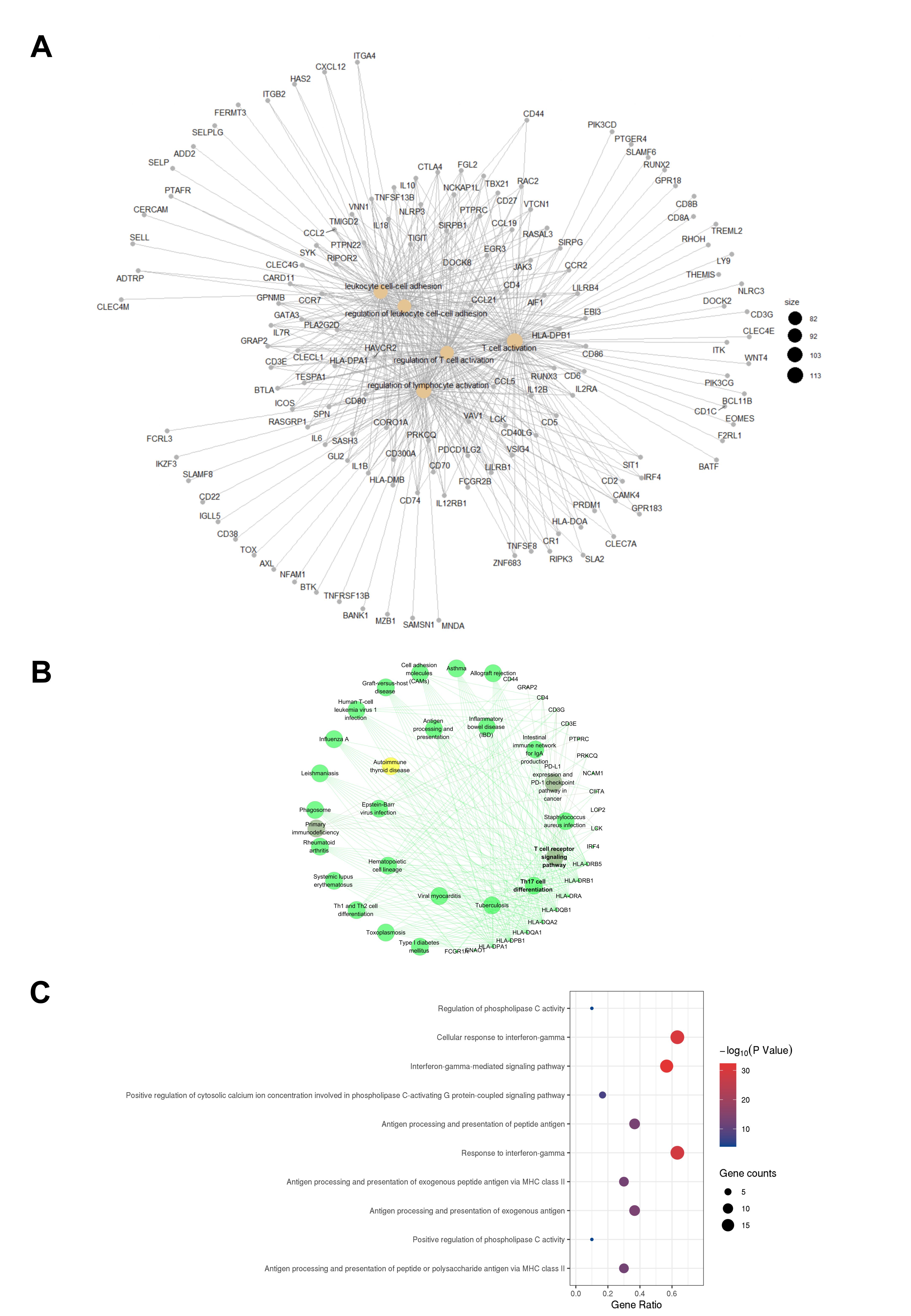

Supplement: Supplementary file 3 [file Image_2.JPEG]

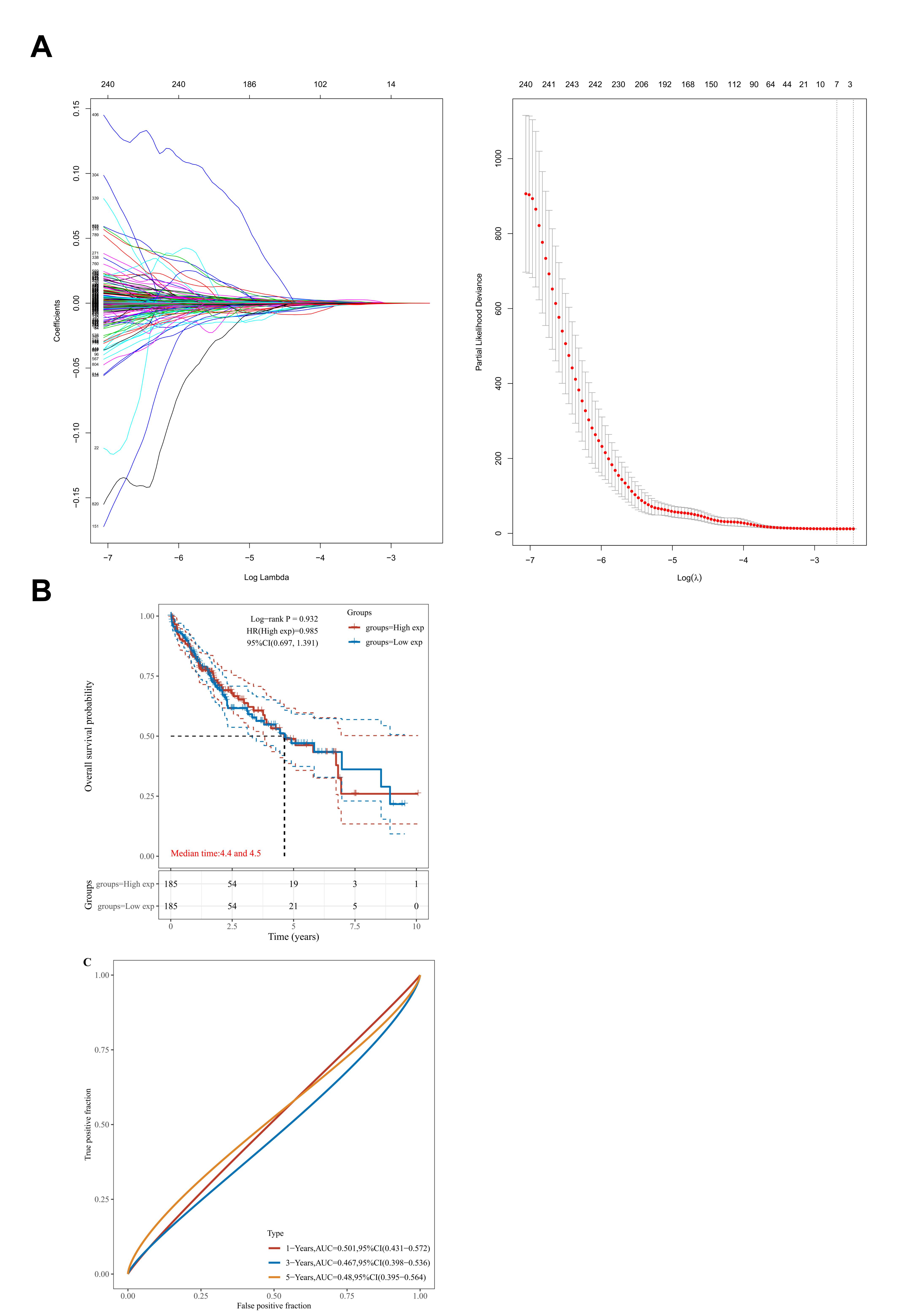

Supplement: Supplementary file 4 [file Image_3.JPEG]
